# Quantitative PCR Effectively Quantifies Triazole-Susceptible and Triazole-Resistant *Aspergillus fumigatus* in Mixed Infections

**DOI:** 10.3390/jof8111120

**Published:** 2022-10-25

**Authors:** Agustin Resendiz-Sharpe, Wannes Van Holm, Rita Merckx, Martine Pauwels, Wim Teughels, Katrien Lagrou, Greetje Vande Velde

**Affiliations:** 1 Department of Imaging and Pathology, Biomedical MRI, KU Leuven, 3000 Leuven, Belgium; 2 Department of Oral and Health Sciences, KU Leuven, 3000 Leuven, Belgium; 3 Department of Microbiology, Immunology and Transplantation, KU Leuven, 3000 Leuven, Belgium; 4 Department of Laboratory Medicine and National Reference Center for Mycosis, Excellence Center for Medical Mycology (ECMM), University Hospitals Leuven, 3000 Leuven, Belgium

**Keywords:** *Aspergillus fumigatus*, triazole-resistance, qPCR, mixed infections

## Abstract

Increasing resistance to triazole antifungals in *Aspergillus fumigatus* is worrisome because of the associated high mortality of triazole-resistant *A. fumigatus* (TRAF) infections. While most studies have focused on single triazole-susceptible (WT) or TRAF infections, reports of TRAF cases developing mixed WT and TRAF infections have been described in several studies. However, the prevalence of mixed infections and their responses to current recommended therapies are unknown and could be inappropriate, leading to poor clinical outcomes. To address the urgent need for tools to diagnose, monitor disease development and therapy efficacies in mixed infection settings where quantification of WT versus TRAF is key, this study developed a novel qPCR assay to differentiate WT and TRAF harboring the *cyp51A*-TR_34_/L98H mutation. The proposed assay successfully quantified *A. fumigatus* and discriminated TRAF-TR_34_ in vitro and in vivo, which was achieved by increasing the yield of extracted DNA through improved homogenization and specific primers targeting the WT-sequence or TR_34_-insertion and a TaqMan-probe directed to *A. fumigatus*. The here-developed qPCR assay overcomes sensitivity issues of methodologies such as CFU counts, providing specific, reproducible, and reliable quantitative information to study and follow up the (interplay and individual) effects of mixed *A. fumigatus* infections on disease development and treatment responses.

## 1. Introduction

The alarming increase in resistance to the recommended initial triazole antifungals therapy in *Aspergillus fumigatus* infections and its associated increased mortality in patients (invasive aspergillosis) urgently calls for better insight into their development and treatment [1,2,3,4]. *A. fumigatus* invasive infections commonly develop in susceptible patients, secondary to the inhalation of airborne spores from the environment [5]. Because of the presence of triazole-susceptible (WT) and triazole-resistant (TRAF) *A. fumigatus* spores in the environment, (it is conceivable that) patients can inhale both types of spores and may develop a triazole-susceptible and -resistant coinfection. However, because of the limited studies and research tools, the prevalence and effects of these mixed infections on disease development, fungal burden dynamics (kinetics and infection ratio-responses), the interplay of susceptible and resistant strains particularly under triazole treatment pressure, and whether current recommended antifungal therapies or treatment modifications lead to appropriate clinical outcomes are not fully known [6].

The occurrence of mixed triazole-susceptible and -resistant *A. fumigatus* infections has been described in a few clinical studies using agar-based or molecular methodologies (qPCR) [7,8,9,10]. A recent clinical study from India indicated concomitant detection of WT and TRAF in 97% (32/33) of the cases through qPCR [10]. Likewise, a study from a tertiary care center in Belgium reported that among all triazole-resistant cases, 40% of patients presented with positive WT cultures within the study periods [11]. These studies suggest that mixed *A. fumigatus* infections are regularly found in patients, yet due to limited information, it is not possible to make any conclusions. So far, preclinical and clinical studies on triazole-resistance have focused mainly on studying single (mono) *A. fumigatus* infections (WT or TRAF) [2,3,6,12,13,14,15,16]. To the best of our knowledge, only one study has focused its efforts on mixed *A. fumigatus* infections, mainly on the fitness of less commonly reported *cyp51A* mutations (main mechanism conferring triazole-resistance) [12], leaving most of the above raised concerns on mixed infections unanswered. Hence, more insight into the development and treatment of mixed infections is urgently needed.

The study of mixed infections must detect the individual and collective effects of susceptible and resistant *A. fumigatus* on disease development and treatment responses compared with single infections, especially under triazole treatment pressure. Thus, the establishment of a quantitative diagnostic tool in preclinical and clinical settings to study and follow up on mixed infections is crucial. Such a tool will gather important information on whether mixed fungal burdens (WT and TRAF ratios) confer differences in disease development compared with single infections, and what effects each phenotypic group may have on one another (synergetic, inhibitory, or none-independent growth). Moreover, it can provide insight into individual WT and TRAF responses to current and novel antifungal therapies (screening studies) and potentially inform personalized and optimal treatment decisions in each patient throughout the disease progression.

The gold-standard technique for studying fungal burden in single-infection samples is through colony-forming unit (CFU) counts in agar plates. However, CFU counts are not suitable for studying mixed *A. fumigatus* infection as they will not provide information on whether the observed growth is due to susceptible or resistant *A. fumigatus* strains. Moreover, limitations such as inherent variability and reproducibility, lower detections, the existence of no culturable cells, and the fragility of the *A. fumigatus* hyphae during sample preparation (rupture) alter the number of cells for CFU counts disputing fungal burden assessment by this technique [10,17,18,19]. Even so, one could envisage a possible solution by employing agar plates with triazole-antifungals where only TRAF strains in mixed infection groups would grow. Nonetheless, this was not successful in our laboratory due to variable CFU counts from expected ratios (±2 log difference) and false-positive growth of WT strains in itraconazole-containing agar plates. The latter is possibly due to in vivo sample characteristics that impeded direct fungal contact with the antifungal. Therefore, only molecular techniques have the potential to overcome this sensitivity issue, namely the quantitative qPCR method with specific TaqMan probes binding to WT and TRAF sequences. The quantification of fungal burden with qPCR in samples from single-infection settings has been previously performed in *A. fumigatus* using single and multicopy genes [12,20,21,22]. However, none have been established for the quantification of WT and mutated *cyp51A*-TR_34_/L98H TRAF (the most commonly reported resistance mechanism), pivotal to a better understanding of mixed infections [23]. This method may further provide a sensitive assay to detect relevant proportions for the WT and TRAF found in real-life infection settings, which is not fully known, and to determine whether these ratios may mimic TRAF environmental prevalences or differ per patient. Hence, the presented study aimed to provide a sensitive, quantitative qPCR assay to enumerate, differentiate, and follow up the WT and mutated *cyp51A* sequences of TRAF harboring the TR_34_/L98H mutation in both clinical and research settings.

## 2. Materials and Methods

### 2.1. Strains, Cultures, and Sample Preparation

The strains used were two triazole-susceptible *A. fumigatus* strains (Af_luc_OPT__red_WT and CBS 144.89) and one triazole-resistant strain (Af_luc_OPT__red_TR34) harboring the *cyp51A* TR_34_/L98H gene mutation [23]. Each strain was grown on Sabouraud’s dextrose agar medium with chloramphenicol (BD, Franklin Lakes, NJ, USA) for three days at 37 °C and harvested by adding 5 mL of distilled water with 0.1% Tween 80 (Sigma-Aldrich, St. Louis, MO, USA) and gently scraping off colonies from the surface with a sterile cotton swab. The collected suspension was vigorously vortexed and filtered (11 μm nylon net filter; MerckMillipore, Burlington, MA, USA) to remove the hyphae or spore clumps. Spore suspensions were subsequently centrifuged, washed and reconstituted in a saline solution. Lastly, the spores were counted using a Neubauer hemocytometer and aliquoted according to the experimental needs.

### 2.2. DNA Extraction

Fungal DNA was extracted using the DNeasy Plant Mini Kit (Qiagen, Dusseldorf, Germany) according to the manufacturer’s instructions, with adaptations of the initial steps. The first step was to determine whether additional homogenization processes could increase the yield of DNA extracted from the fungal cells. Three previously described fungal homogenization methodologies were adapted and compared for their effects on DNA yield extraction through qPCR [24]. In all of the methods, samples (conidia suspension in 2 mL of saline solution) were initially homogenized using the gentleMACS M tubes™ and dissociator™ (program RNA 2; Miltenyi-Biotec, Bergisch Gladbach, Germany), followed by centrifugation and supernatant removal.

Method A consisted of fungal pellets reconstitution with 360 µL of AL buffer (cell lysis solution; Qiagen, Dusseldorf, Germany) and 20 µL proteinase K (20 mg/mL; Qiagen, Dusseldorf, Germany) incubated for 2 h at 55 °C. The samples were then transferred to bead-containing tubes (MP lysing matrix E tubes; MP biomedicals, Santa Ana, CA, USA) and shaken in an MP Biomedical FastPrep-24 instrument for 60 s (speed 6 m/s). In method B, pellets were reconstituted in 600 µL of Sorbitol buffer [24] and 20 µL proteinase K (incubation of 2 h at 55 °C), with subsequent second bead-beating homogenization, as referred to in method A. Method C consisted of pellet reconstitution in 600 µL of Sorbitol buffer and 150 µL of lyticase (300 U; Sigma Aldrich, Burlington, MA, USA) with 30 min of incubation at 30 °C. Next, the samples were incubated with proteinase K (methods A and B), followed by bead-beating homogenization (60 s each) twice with 5 min ice incubation after each round. Afterward, fungal DNA extraction was performed using the Qiagen mini plant kit according to the manufacturer’s recommendation. The quality and amount of extracted DNA were assessed by spectrophotometry (A260/A280 and A260/A230 ratios; Nanodrop 2000 Thermo-Fisher Scientific, Waltham, MA, USA). Each experiment was repeated independently three times from three different extracted DNA samples and run-in duplicate by two operators with varying levels of technical expertise.

### 2.3. qPCR Reaction and Calculations

**Figure 1:**
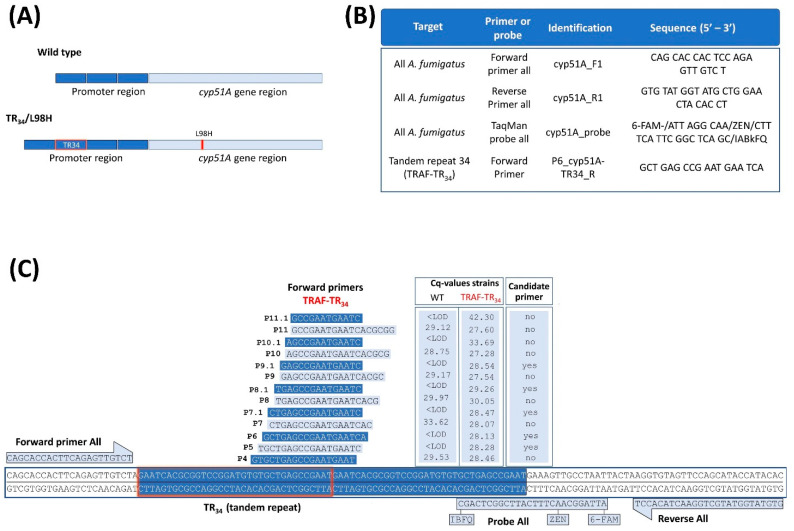
Quantitative PCR assay targeted to the detection of the single *cyp51A* copy gene and the mutated tandem repeat 34 (TR_34_). (**A**) Schematic comparison of the *cyp51A* gene and its promoter region of a WT and a triazole-resistant *A. fumigatus* harboring a TR_34_/L98H *cyp51A* gene mutation (TRAF-TR_34_). (**B**) Selected PCR primers and probe sequences for our qPCR assay targeted to detect the single *cyp51A* copy gene in all *A. fumigatus* strains and strains with mutated TR_34_. (**C**) Oligonucleotide sequences and primer map locations of 13 forward primers targeted to the tandem repeat 34-region of TRAF-TR_34_ strains. Cq reaction values and their specificity for TR_34_ detection are depicted in the graphic table on the right (1 × 10^6^ WT and TRAF-TR_34_ conidia). Candidate primers for mutant detection were selected if they amplified the mutant TRAF-TR_34_ (Cq ± 28) without amplifying the WT (<LOD). Abbreviations: quantification cycle (Cq), below limit of detection (<LOD), wild-type A. fumigatus (WT), triazole-resistant A. fumigatus harboring the TR_34_/L98H mutation (TRAF-TR^34^). Cq value represents the average of three reactions (1 × 10^6^ conidia per sample). Figure 1C primers were designed using SnapGene (www.snapgene.com (accessed on 1 April 2022)).

All qPCR assays targeting the single copy *cyp51A* gene in all *A. fumigatus* or the mutated *cyp51A* tandem repeat 34-region of TRAF-TR_34_/L98H strains (Figure 1A) were done using a CFX96 real-time PCR system (Bio-Rad, Hercules, CA, USA), and were analyzed with its associated CFX Manager software (version 3.1). The TaqMan probe-based qPCR reaction was performed using 12.5 µL of Takyon Rox probe master mix dTTP blue (Eurogentec, Liège, Belgium), 0.5 µL of each primer (final concentration 900 nM; ordered from IDT, Leuven, Belgium), 0.5 µL probe (final concentration 200 nM; IDT, Leuven, Belgium), 5 µL of the template, and 6 µL of nuclease-free water (Invitrogen, Waltham, MA, USA).

Cycle conditions were as follows: initial step at 50 °C for 2 min and 95 °C for 10 min, followed by 45 cycles of 95 °C for 15 s and 60 °C for 1 min. PCR primers (cyp51A_F1/R1) and probe (cyp51A_probe) sequences were used to amplify the single copy *cyp51A* gene of all *A. fumigatus* (WT and TRAF-TR_34_ strains; Figure 1B) and the probe sequence for the tandem repeat 34-region of TRAF-TR_34_ strains (TaqMan cyp51A_TR_34__probe), were selected from van der Linden et al. [25]. Of the 20 designed primers, 13 forward primers were tested to select the primer with specific binding within the tandem repeat 34-*cyp51A* promoter region of TRAF- TR_34_ strains (only mutant detection) and without aspecific binding outside the tandem repeat (no WT detection) (Figure 1C). Primers were designed in SnapGene (version 5.0.5) based on reference strain AF 338659 and TR_34_/L98H *A. fumigatus* V052-35 strain [25,26].

The gene copy number was determined by running standard curves in conjunction with each set of analyzed samples. The quantification of fungal DNA in each sample was inferred based on generated threshold cycle values (Cq) of standard curves from a six 1:10 dilutions (log_10_) from isolated WT or TRAF-TR_34_ fungal DNA suspensions (1 × 10^8^ conidia). The samples’ Cq values were plotted against those of the standard curve DNA template and were subsequently converted into copy numbers. Fungal burden quantification (copy numbers) was reported as the conidia equivalent (log_10_ CE) per sample (x/mL; x/gram tissue). In addition, *A. fumigatus* conidia suspensions were cultured and enumerated (CFUs) at 48 h to corroborate fungal concentration. Only standard curves with a demonstrated r^2^ value of >0.97 were used for the analysis. Each extracted sample (different days) was run in triplicate to test reproducibility likewise by two operators with different levels of technical expertise; their average mean was used for further study.

### 2.4. Galleria Mellonella Infection

For in vivo analysis, healthy *G. mellonella* larvae were selected and divided into six groups (*n* ≤ 18; 3 per group); the larvae in groups 1–5 were infected and larvae in group 6 were used as uninfected controls (sham-saline solution). Each larva from the infection groups (1–5) was inoculated with a total of 100,000 spores (1 × 10^5^) with either susceptible, triazole-resistant, and both *A. fumigatus* conidia ratios (log difference) in 10 µL through the last left proleg using a syringe. After one hour of infection, larvae were pooled together and homogenized (Omni tissue homogenizer; Omni International, Tulsa, OK, USA) in 600 µL of saline solution. DNA extraction was subsequently performed as mentioned above.

### 2.5. Statistical Analysis

Statistical analysis was done using GraphPad Prism version 9.3.1. The one-way analysis of variance (ANOVA; alpha ≤ 0.05) with multiple comparison analysis (Sydak’s correction) were used to determine statistically significant differences between groups. Pearson correlation coefficients were used for the correlation analyses of the variables.

## 3. Results

### 3.1. Increased DNA Extraction Yield through Optimized Sample Homogenization Methodology

**Figure 2:**
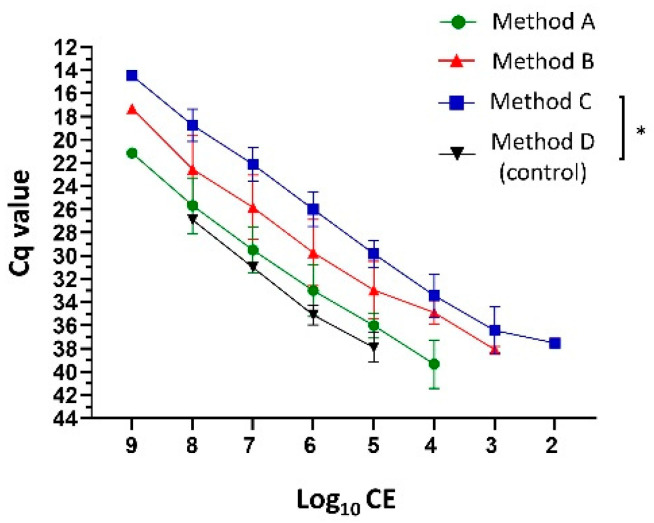
Cycle threshold (Cq) values relationship and comparison between the number of *A. fumigatus* conidia and the DNA extraction methodologies. Conidial Equivalents (CE) determination according to the obtained Cq values from the log_10_ dilution from the extracted DNA (1 × 10^9^ wild-type *A. fumigatus* conidia; cyp51A_probe) without additional (method D; Qiagen Mini Plant Kit protocol) and with additional preceding homogenization methodologies (Method A–C). Data lines represent the mean value ± standard deviation (SD) of the results from multiple samples (*n* ≤ 3). * *p* ≤ 0.05, to control (method D; one-way repeated measures ANOVA with multiple comparison analysis; Sidak’s correction).

Typically, the yield of extracted DNA from fungal cells is less compared with bacteria due to the fungal cell wall structural complexity. Low initial DNA amounts can result in insufficient amplification. The inclusion of various homogenization processes has been reported to increase the yield of DNA extracted from the fungal cells. Here, the combinations of different sample homogenization methodologies (chemical, enzymatic, and mechanical; Methods A–C) before DNA extraction were compared in order to determine whether this would increase the yield of fungal burden detection by qPCR using primers (cyp51A_F1 and cyp51A_R1) and probe sequences (cyp51A_probe and cyp51A_TR_34__probe) [25]. Extraction Method C (sorbitol buffer, lyticase, proteinase K, and bead beating) provided the most sensitive range limit of log_10_ CE detection from log 9 to log 2 (Figure 2; 1 × 10^9^ starting conidia). Method C was followed by Method B (sorbitol buffer, proteinase K, and bead beating) from log 9 to log 3 and Method A (AL buffer, proteinase K, bead beating) from log 9 to log 4. This resulted in four (*p* ≤ 0.049), three, and two additional log detections compared with no additional homogenization (Method D), respectively, in both WT and TRAF-TR_34_/L98H *A. fumigatus* conidia (cyp51A_probe). Likewise, Method C provided the lowest Cq values and lowest variation (log 8 Cq-average of 18.74 ± 1.39 (SD) compared with 25.67 ± 2.39 (method A) and 22.53 ± 2.88 (method B)) among all of the tested methodologies and was thus used as our standard DNA extraction methodology.

### 3.2. Selection of Specific Primers for the Mutated TR_34_-bp Insertion in A. fumigatus

Unexpectedly, the specificity of the probe for the mutated TR_34_-bp repetitive insertion (cyp51A_TR_34__probe) from van der Linden et al. [25] could not be confirmed, as signals (Cq values) in samples containing only WT *A. fumigatus* were detected. These signal detections averaged 2 logs lower compared with the reactions with the cyp51A_probe (all detecting *A. fumigatus*), most likely due to aspecific binding. This aspecific detection of signals represents an issue for accurate quantification, particularly in mixed infection settings. Therefore, new specific forward primers were designed to target the mutated TR_34_-bp repetitive sequence to only amplify TRAF-TR_34_ (Figure 1B) and were combined with the general reverse primer and general *A. fumigatus* probe detection (cyp51A_R1 primer, cyp51A_probe; Figure 1C). Of the 20 designed primers, 13 TR_34_-forward primers showed low in silico unspecific binding outside the tandem repeat and were tested further (Figure 1B). Using the following criteria, five forward primers, namely P9.1, P8.1, P7.1, P6, and P5, were selected as candidate primers (Figure 1B). These primers showed no amplification of wild-type DNA (Cq values below the limit of detection (LOD)) and showed a similar Cq to the standard representing 1 × 10^6^ conidia (Cq’s ranging from 28.13–29.26). Of these five, primer P6 provided the most significant CE correlation (r ≤ 0.9939; *p* ≤ 0.0001) to the initial conidia in the sample with accurate reproducibility (Cq coefficient of variation < 1.0%) and least variability (SD), with no signal detections in the WT samples (<LOD). For this, primer P6 was thus selected as the mutant forward primer. The limit of detection of this assay ranged from log 9 to log 2 (minimum ≤ 118 CE copy numbers), yet below 1 × 10^3^ were not always detected. The qPCR reaction characteristics and CE copy number determination for all *A. fumigatus* and TRAF-TR_34_ *A. fumigatus* using the P6 forward primer are shown in Table 1.

### 3.3. Mixed Triazole-Susceptible and Resistant A. fumigatus Detection and Ratio Quantification

**Figure 3:**
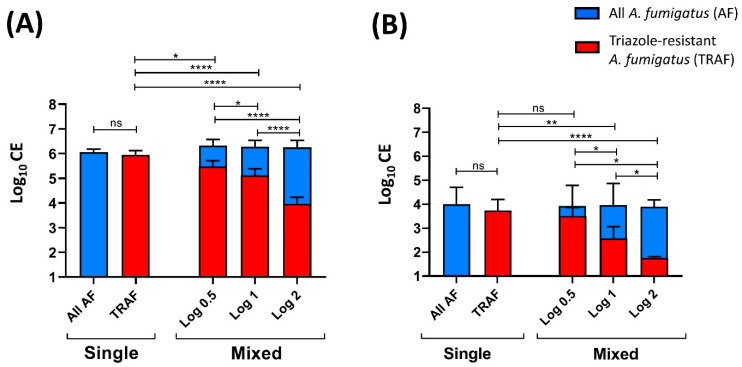
Quantitative ratio determination of triazole-resistant *A. fumigatus* in mixed susceptible and resistant Aspergillus samples. (**A**) In vitro quantification (conidial equivalents (CE)) of all *A. fumigatus* (AF) and of triazole-resistant AF (TRAF; TR_34_/L98H) ratios in single and mixed triazole-susceptible and triazole-resistant log ratios samples (0.5, 1, and 2 log_10_ difference; final 1 × 10^6^ spores). (**B**) In vivo CE log ratio quantification of infected *Galleria mellonella* larvae homogenates with single and mixed log ratios inoculum of triazole-susceptible and resistant conidia (0.5, 1, and 2 log_10_ difference; final inoculum of 1 × 10^5^ spores). Data are mean ± standard deviation (SD) of the results from multiple samples (*n* = 9 (**A**), *n* = 6 (**B**)). * *p* ≤ 0.05, ** *p* ≤ 0.005, **** *p* ≤ 0.0005 to single TRAF control (one-way repeated measures ANOVA) and between ratios (multiple comparison analysis; Sidak’s correction). ns: not significant.

Subsequently, the capabilities of this qPCR assay to determine and differentiate the number of TRAF-TR_34_ CE present in mixed WT and TRAF-TR_34_ *A. fumigatus* samples in vitro and in vivo were determined. For the in vitro assay, mixed *A. fumigatus* samples containing a log difference decrease of 0.5, 1, or 2 of TRAF-TR_34_ from WT conidia numbers (1 × 10^6^ conidia in total [log 6], Figure 3A) were analyzed and compared. Comparable detections of all *A. fumigatus* (log 6.3 ± SD 0.13) in all samples were observed, with no significant differences between single and mixed TRAF-TR_34_ ratio-sample groups. In the WT only samples, the use of the TRAF-TR_34_ forward primer resulted in no or below the LOD signals (Cq values), confirming the specificity of this primer for only TRAF-TR_34_. In mixed samples, there was a significant log-dependent decrease in CE numbers in accordance to the sample TRAF-TR_34_ ratios compared with the single TRAF-TR_34_ samples (log 0.5 *p* ≤ 0.022, log 1 *p* ≤ 0.0001 and log 2 *p* ≤ 0.0001; Figure 3A) and with the obtained CE log numbers from all *A. fumigatus* within the same sample (log 0.5 *p* ≤ 0.023, log 1 *p* ≤ 0.0001 and log 2 *p* ≤ 0.0001). Significant CE differences between ratios were likewise present between mixed log ratio samples (log 0.5 vs. log 1 *p* ≤ 0.0308, log 0.5 vs. log 2 *p* ≤ 0.0001 and log 1 vs. log 2 *p* ≤ 0.0001, Figure 3A).

To validate this qPCR assay in vivo, a *Galleria mellonella* larvae model of aspergillosis was used. Larvae groups were infected with either single (WT or TRAF-TR_34_) or mixed *A. fumigatus* inoculums (Figure 3B). Mixed inoculums contained a mixture of WT and three different concentrations of TRAF-TR34 conidia (0.5, 1, or 2 log lower conidia than WT; final mixed-sample count of 1 × 10^5^ conidia). Likewise, the detection of all *A. fumigatus* was comparable among all infected larvae, regardless of the inoculated single or mixed TRAF-TR_34_ ratios (4 ± 0.61 log CE). On average, the mean CE numbers were one log lower compared with the inoculated conidia numbers with a higher variation (SD) compared with the in vitro findings. As anticipated, using the TRAF-TR_34_ primer, no detections were observed in the WT only infected larvae, verifying the specificity. The TRAF-TR_34_ quantification of mixed infected larvae resulted in an inoculum-dependent log decrease in CE numbers compared with only the TRAF-TR_34_ infected larvae (log 1 *p* ≤ 0.005 and log 2 *p* ≤ 0.0001). Similarly, a log-dependent decrease in TRAF-TR_34_ CE numbers from total *A. fumigatus* within the same sample was observed (log 1 *p* ≤ 0.0106 and log 2 *p* ≤ 0.0001). Differences between the total CE numbers from TRAF-TR_34_ only and within infected groups to TRAF-TR_34_ CE of mixed log 0.5 infected larvae were also observed, yet they were not large enough to achieve statistical significance. TRAF-TR_34_ log ratio differences among mixed infected larvae were also significantly detected (log 0.5 vs. log 1 *p* ≤ 0.024, log 0.5 vs. log 2 *p* ≤ 0.016, and log 1 vs. log 2 *p* ≤ 0.048).

## 4. Discussion

Overall, the mechanisms involved in the disease development of *A. fumigatus* mixed infections with susceptible and resistant isolates are poorly understood. However, the presence of diverse isolates with different susceptibility profiles complicates the management of mixed infections and the survival of patients. Here, we provide a novel qPCR approach that quantifies *A. fumigatus* (CE) in in vitro and in vivo settings and successfully discriminates TRAF harboring the TR_34_/L98H *cyp51A* gene mutation from triazole-susceptible ones (WT) in a quantitative manner.

The establishment of a powerful quantitative diagnostic tool to study and follow up on mixed aspergillus infections is crucial to gather important information on their disease development compared with single infections and their individual progression and responses to current and novel antifungal therapies (screening studies). Currently, various qPCR assays have been used in the study and diagnosis of WT and TRAF; however, they mainly focused on detecting and identifying these fungi. Some assays use multi-locus genes such as the 18S or 28S rRNA, and others on single-copy genes such as *FKS, Beta tubulin, PyrG*, and *ARG4* [20,21,22,27]. Assays using multi-locus genes are suitable for fungal detection and identification, but not for accurate quantitative fungal burden determination as they provide several copy numbers of the same gene. In contrast, single-copy genes are recommended in order to obtain equivalent fungal cell nuclei to copy number ratios (1:1), and genes such as FKS1 have been usefully used in vivo studies [20,22]. However, none of these gene assays are convenient for studying triazole-resistant infections secondary to *cyp51A* gene mutations. The few described *cyp51A* gene qPCR assays have directed their efforts mainly to diagnostic purposes through melting curve analysis (fluorescent peak ratios of discriminative probes) or to the quantification of less commonly reported *cyp51A* gene mutations such as the M220K [8,12,25,28,29]. However, to the best of our knowledge, none had successfully quantified the fungal burdens of the most commonly reported TRAF-TR_34_/L98H mutation and WT sequences in samples simultaneously. Therefore, the use of the here described groundbreaking assays will provide relevant quantitative information for the study of mixed triazole-susceptible and resistant *A. fumigatus* infections.

To accomplish this, adequate yields of extracted fungal DNA are necessary for optimal quantification. However, fungal DNA extraction is challenging, most likely due to their cell wall resistance to most lysing methods. This resistance results in reduced fungal DNA release and the need for larger amounts of sample or tissue, which are not always available. To overcome these issues, several fungal homogenization and DNA extraction protocols have been described using PCR, with variable outcomes [24,30]. Thus, to increase the yield of extracted DNA for accurate fungal burden detection by qPCR, several pre-treatment methodologies combining different described chemical, enzymatic, and mechanical homogenizations were evaluated (Methods A–C) [20,24]. The use of liquid nitrogen to ground fungal-containing samples before DNA extraction was not included, as it did not provide additional benefits to traditional methods in our laboratory in the past [24,27]. Of all of the tested methodologies, Method C provided the best reproducible extraction yield, DNA quality, and qPCR results (triplicates of three different samples). This method consisted of sample-pellet reconstitution and incubation in a Sorbitol buffer and lyticase followed by a second incubation with proteinase K and a two-bed beating homogenization with ice-incubation between homogenizations to facilitate protein precipitation. Each step confers specific functions that either target the fungus cell wall or protect and favor DNA recovery. Sorbitol is an osmotic agent that presumptively removes cellular contaminants within the cytosol, such as polysaccharides and polyphenols, which co-precipitate or bind irreversibly to DNA, affecting its extraction [31]. Lyticase catalyzes fungal cell wall lysis by β-1,3-glucanase activity and by participating in spheroplasts formation [24,32]. Proteinase K assists in the removal of proteins, particularly nucleases that may affect the DNA present in samples [24,32]. Method A (AL buffer, proteinase k, and beat beading) provided the least desirable results. This methodology presented with excessive foam and restricted liquid material that limited sample collection, which could partially explain these results. Nevertheless, it performed better than no additional homogenization. The AL buffer (containing guanidinium chloride) interfered with polar components such as hydrogen bonds, promoting DNA release by cell lysis and protein denaturation [33].

The aim of the proposed qPCR assay is to discriminate triazole-resistant *A. fumigatus* harboring the TR_34_/L98H *cyp51A* gene mutation from triazole-susceptible ones (WT) in a quantitative manner. However, the initially used primers and probe sequences (cyp51A_F1/R1 primers, cyp51A_probe, and cyp51A_TR_34__probe [25]) targeting the 34-bp tandem repeat in TRAF-TR_34_ had inadequate results. The detection of the total number of *A. fumigatus* independent of *cyp51A* mutations, necessary for the ratio determination of TRAF-TR_34_, was successfully achieved using the cyp51A_probe. This setup provided a significantly large positive correlation (r ≤ 09893; *p* ≤ 0.001; data Table 1) between the initial sample amount and obtained CE by qPCR in vitro, in both triazole-susceptible and -resistant *A. fumigatus* samples, with adequate reproducibility (Cq coefficient of variation) and reduced variability (SD). However, this was not the case when using the specific probe for the mutated TR_34_-bp insertion (cyp51A_TR_34__probe), as signals were obtained from DNA extracted from samples containing only WT *A. fumigatus*. The probe from van der Linden and colleagues targeting the TR_34_ insertion binds to the first 9 bp of the repetitive insertion (22 bp long), with 13 bp overlapping with the WT sequence. The primers used were located outside the mutant sequence, allowing for the amplification of the WT sequence, resulting in a large number of copies through PCR amplification. As a consequence, large amounts of WT copies were produced and any slight unspecific binding of the probe may result in an unwanted signal. Therefore, instead of using this aspecific probe, several mutant-specific forward primers were effectively designed to limit DNA amplification, only in the presence of the TR_34_ insertion sequence to avoid aspecific signals; in combination with the already validated cyp51A_R1 reverse primer and cyp51A_probe (all/total *A. fumigatus* assay). Of the mutant-specific primers, primer P6 provided the most significant, reproducible, and least variable TRAF-TR_34_ CE correlation (r ≤ 0.9939; *p* ≤ 0.0001) with no signal detections in the WT samples (<LOD).

The limit of detection of this assay ranged from log 9 to log 2 CE, yet anything below log 3 CE was not always detected. Likewise, the signal above log 8 CE was sometimes variable and lower than expected. The observed variability may be due to the saturation of the binding capacity of the membrane kit used for extraction, increased cellular debris (RNA and proteins), or salt precipitates (A_260_/A_280_ ratio < 1.7). Therefore, the conidia range that could be reliably quantified by our method is from log 8 to log 3, which is comparable to other *A. fumigatus* qPCR single-gene assays [20,22].

The methodology used effectively quantified and determined different log ratios (0.5, 1, and 2) of TRAF-TR_34_ within mixed samples in vitro, which were correlated to the total *A. fumigatus* present in the samples with reproducible results. Likewise, log ratio differences of TRAF-TR_34_ could be determined in vivo using *G. mellonella* larvae infected with mixed log-ratio inoculums (0.5, 1, or 2). Compared with in vitro conditions, the mean CE numbers in infected larvae presented a slightly increased variation and were one log less compared with the inoculated conidia numbers. This observed difference was expected, to some extent, due to the increased available homogenated sample amount. Likewise, the existence of components present in the larvae homogenates that interfere or compete with fungal DNA extraction may play a role. However, compared with the total *A. fumigatus* numbers within larvae groups, log ratio differences were still significantly detected in TRAF-TR_34_ log 1 and log 2 infected larvae with reproducible results and, to a lesser extent, in the log 0.5 ones. The detection of CE of <0.5 log was not possible as the Cq values of TRAF-TR34 and WT *A. fumigatus* were too close to differentiate.

It should be noted that this new assay was designed specifically for binding within the TR_34_ region of the strain used. Yet, the *cyp51A* gene region was very conserved within *A. fumigatus*. Therefore, the occurrence of minor differences in nucleotides between cyp51A-TR_34_/L98H strains that might influence the efficiency of this new assay are unlikely [34]. Similarly, the efficacy of the assay could be compromised in the event of multiple TR_34_ insertions within the same strain (infrequently reported). In this scenario, melt curve analysis or agarose gel electrophoresis could be used to evaluate the presence of multiple amplicons and their effects on the assay, but this was not performed due to such a strain being unavailable for the current study.

In conclusion, the methodology developed in this study offers a powerful tool for studying triazole-resistant *A. fumigatus* (TR_34_/L98H) infections and their treatment, particularly in much-needed areas such as mixed triazole-susceptible and -resistant infections, which are still poorly understood. This assay has the potential to procure relevant quantitative information on fungal burden dynamics (kinetics and infection ratio-responses) and the interplay of WT and TRAF-TR_34_ mixed infections at several stages of disease development in preclinical and clinical scenarios. The provided assay can shed light on different clinical situations of mixed infections, such as the prevalence (in patients and within clinical samples), their development in patients already receiving antifungal therapy, and whether current recommended antifungal treatments lead to appropriate clinical outcomes. In addition, the assay could be potentially used as an initial colonization screening in patients at risk of developing aspergillosis (e.g., critically ill or chronic pulmonary diseases) in order to create awareness among clinicians regarding the possibility of future TRAF-TR_34_/L98H infection development that can provide adequate sensitivity. To this end, further research on the assay’s characteristics performance in human tissue samples, such as bronchoalveolar lavage fluid or biopsy materials, is the required next step. This assay likewise provides the unique opportunity to assess, in vitro and in vivo, current and novel antifungal treatment responses to mixed *A. fumigatus* infections and the evolution of the disease upon diverse fungal burden rates and antifungal therapies, particularly in scenarios receiving triazole antifungals.

## Figures and Tables

**Table 1:** qPCR reaction characteristics and determination of the conidia equivalent copy number of the predicted specific single copy *cyp51A* gene.

		All *A. fumigatus* Primers				TRAF-TR_34_ *A. fumigatus* Primers			
Sample Phenotype	Conidia in Sample	Cq Average ± SD	Cq (% CV)	Average Log_10_ (x/mL) ± SD	Conidia Equivalent (No.)	Cq Average ± SD	Cq (% CV)	Average Log_10_ (x/mL) ± SD	Conidia Equivalent (No.)
WT	1 × 10^8^	21.91 ± 0.15	0.66	7.78 ± 0.14	6.87 × 10^7^	ND	ND	ND	ND
WT	1 × 10^7^	24.35 ± 0.11	0.46	7.07 ± 0.04	1.20 × 10^7^	ND	ND	ND	ND
WT	1 × 10^6^	27.68 ± 0.09	0.32	6.07 ± 0.03	1.20 × 10^6^	ND	ND	ND	ND
WT	1 × 10^5^	31.59 ± 0.15	0.47	4.90 ± 0.05	7.87 × 10^4^	ND	ND	ND	ND
WT	1 × 10^4^	34.91 ± 0.30	0.85	3.90 ± 0.09	7.98 × 10^3^	ND	ND	ND	ND
WT	1 × 10^3^	37.66 ± 0.35	0.92	3.07 ± 0.10	1.18 × 10^3^	ND	ND	ND	ND
WT	1 × 10^2^	ND	ND	ND	ND	ND	ND	ND	ND
TRAF-TR_34_	1 × 10^8^	21.72 ± 0.16	0.73	7.83 ± 0.05	7.14 × 10^7^	22.02 ± 0.17	0.79	7.74 ± 0.07	5.68 × 10^7^
TRAF-TR_34_	1 × 10^7^	24.02 ± 0.05	0.22	7.14 ± 0.02	1.42 × 10^7^	24.27 ± 0.10	0.43	7.07 ± 0.02	1.19 × 10^7^
TRAF-TR_34_	1 × 10^6^	27.92 ± 0.16	0.59	5.90 ± 0.05	8.82 × 10^5^	27.30 ± 0.15	0.55	5.88 ± 0.08	8.17 × 10^5^
TRAF-TR_34_	1 × 10^5^	31.62 ± 0.14	0.45	4.75 ± 0.11	6.01 × 10^4^	31.45 ± 0.34	0.97	4.86 ± 0.04	8.45 × 10^4^
TRAF-TR_34_	1 × 10^4^	34.55 ± 0.34	0.97	3.84 ± 0.11	6.89 × 10^3^	34.30 ± 0.23	0.67	4.14 ± 0.07	2.84 × 10^4^
TRAF-TR_34_	1 × 10^3^	37.34 ± 0.37	0.98	2.97 ± 0.11	9.33 × 10^2^	36.88 ± 0.39	0.77	3.25 ± 0.18	2.83 × 10^3^
TRAF-TR_34_	1 × 10^2^	38.90 ± 0.36	0.93	2.48 ± 0.12	3.05 × 10^2^	38.27 ± 0.36	0.93	2.68 ± 0.12	4.83 × 10^2^

**Abbreviations:** Cq (cycle threshold), CV (coefficient of variation), standard deviation (SD), wild-type *A. fumigatus* (WT), triazole-resistant *A. fumigatus* harboring a TR_34/_L98H *cyp51A* gene mutation (TRAF-TR_34)_, none detected (ND). SD represents reactions from three different samples done in triplicates.

## Data Availability

Not applicable.
